# Authors’ response: Mezei et al’s “Comments on a recent case-control study of malignant mesothelioma of the pericardium and the tunica vaginalis testis”

**DOI:** 10.5271/sjweh.3910

**Published:** 2020-12-16

**Authors:** Alessandro Marinaccio, Dario Consonni, Carolina Mensi, Dario Mirabelli, Enrica Migliore, Corrado Magnani, Davide Di Marzio, Valerio Gennaro, Guido Mazzoleni, Paolo Girardi, Corrado Negro, Antonio Romanelli, Elisabetta Chellini, Iolanda Grappasonni, Gabriella Madeo, Elisa Romeo, Valeria Ascoli, Francesco Carrozza, Italo Francesco Angelillo, Domenica Cavone, Rosario Tumino, Massimo Melis, Stefania Curti, Giovanni Brandi, Stefano Mattioli, Sergio Iavicoli, ReNaM Working Group *

**Affiliations:** 1 Occupational and Environmental Medicine, Epidemiology and Hygiene Department, Italian Workers’ Compensation Authority (INAIL), Roma, Italy; 2 COR Lombardy, Epidemiology Unit, Fondazione IRCCS Ca’ Granda Ospedale Maggiore Policlinico, Milano, Italy; 3 COR Piedmont, Unit of Cancer Epidemiology, University of Turin and CPO-Piemonte, Torino, Italy; 4 Department of Translational Medicine, University of Eastern Piedmont, Novara, Italy; 5 COR Liguria, UO Epidemiology, IRCCS Az. Ospedaliera Universitaria San Martino, National Cancer Research Institute (IST), Genova, Italy; 6 COR Province of Bolzano, Alto Adige Health Local Unit, Bolzano, Italy; 7 COR Veneto, Occupational Health Unit, Department of Prevention, Padua, Italy; 8 COR Friuli-Venezia Giulia, University of Trieste -Trieste General Hospitals, Clinical Unit of Occupational Medicine, Trieste, Italy; 9 COR Emilia-Romagna, Health Local Unit, Public Health Department, Reggio Emilia, Italy; 10 COR Tuscany, Cancer Prevention and Research Institute, Unit of Environmental and Occupational Epidemiology, Firenze, Italy; 11 COR Marche, University of Camerino, Hygienistic, Environmental and Health Sciences Department, School of Sciences of the drug and the products of health, Camerino, Italy; 12 COR Umbria, Umbria Region, Department of Prevention, veterinary health and food safety, Perugia, Italy; 13 COR Lazio, Lazio Region, Department of Epidemiology and Department of Radiological, Oncological and Pathological Sciences, Sapienza University, Roma, Italy; 14 COR Molise, Cardarelli Hospital, Oncology Unit, Campobasso, Italy; 15 COR Campania, Second University of Naples, Department of Experimental Medicine, Napoli, Italy; 16 COR Puglia, University of Bari, Department of Interdisciplinary Medicine, Section of Occupational Medicine ‘‘B. Ramazzini’’, Bari, Italy; 17 COR Sicily, Cancer Registry ASP Ragusa and Sicily Regional Epidemiological Observatory, Italy; 18 COR Sardegna. Regional Epidemiological Center, Cagliari, Italy; 19 Department of Medical and Surgical Sciences, University of Bologna, Bologna, Italy; 20 Department of Experimental, Diagnostic and Specialty Medicine, University of Bologna, Bologna, Italy

Mezei et al’s letter (1) is an opportunity to provide more details about our study on pericardial and tunica vaginalis testis (TVT) mesothelioma (2), which is based on the Italian national mesothelioma registry (ReNaM): a surveillance system on mesothelioma, with individual asbestos exposure assessment.

Incidence of pericardial mesothelioma has been estimated around 0.5 and 0.2 cases per 10 million person-years in men and women, respectively, and around 1 case for TVT mesothelioma. ReNaM collected 138 cases thanks to its long period of observation (1993–2015) and national coverage. Conducting a population-based case–control study with incidence-density sampling of controls across Italy and over a 23 year time-span should have been planned in 1993 and would have been beyond feasibility and ReNaM scope. We rather exploited two existing series of controls (3).

The resulting incomplete time- and spatial matching of cases and controls is a limitation of our study and has been acknowledged in our article. The analysis of case–control studies can nevertheless be accomplished in logistic models accounting for the variables of interest, in both individually and frequency matched studies (4). Furthermore, analyses restricted to (i) regions with enrolled controls, (ii) cases with definite diagnosis, (iii) incidence period 2000–2015, and (iv) subjects born before 1950 have been provided in the manuscript, confirming the strength of the association with asbestos exposure (supplemental material tables S4–7).

Following Mezei et al’s suggestion, we performed further sensitivity analyses by restriction to regions with controls and fitting conditional regression models using risk-sets made of combinations of age and year of birth categories (5-year classes for both). We confirmed positive associations with occupational exposure to asbestos of pericardial mesothelioma, with odds ratios (OR) (adjusted for region) of 9.16 among women [95% confidence interval (CI) 0.56–150] and 5.63 (95% CI 1.02–31.0) among men; for TVT mesothelioma the OR was 7.70 (95% CI 2.89–20.5). Using risk sets of age categories and introducing year of birth (5-year categories) as a covariate (dummy variables) the OR were similar: OR (adjusted for region) of 9.17 among women (95% CI 0.56–150) and 5.76 (95% CI 1.07–31.0) among men; for TVT the OR was 9.86 (95% CI 3.46–28.1).

Possible bias from incomplete geographical overlap between cases and controls has been addressed in the paper (table S4) and above. In spatially restricted analyses, OR were larger than in those including cases from the whole country, indicating that bias was towards the null. Mezei et al further noted that “the regional distribution of controls is different from that of person-time observed”. This objection is not relevant because the above analyses were adjusted by region.

Our controls were provided by a population-based study on pleural mesothelioma (called MISEM) and a hospital-based study on cholangiocarcinoma (called CARA). In MISEM, the response rate was 48.4%, a low but not unexpected rate as participation among population controls is usually lower and has been declining over time (5). It is important to underline that ReNaM applied the same questionnaire that was used for interviews and carried out the same exposure assessment as both MISEM and CARA.

As repeatedly stated in ReNaM papers (6–7), each regional operating center assesses asbestos exposure based on the individual questionnaire, other available information, and knowledge of local industries. Occupational exposure to asbestos is classified as definite, probable or possible. Occupational exposure is (i) definite when the subject’s work was reported or otherwise known to have involved the use of asbestos or asbestos-containing materials (MCA); (ii) probable when subjects worked in factories where asbestos or MCA were used, but their personal exposure could not be documented; and (iii) possible when they were employed in industrial activities known to entail the use of asbestos or MCA. Hence, the definite and probable categories are closer to one another and were combined in our analyses. In any case, restricting analyses to subjects with definite occupational exposure and using each set of controls separately, as suggested by Mezei et al, yielded elevated OR for TVT and pericardial mesothelioma among men using both the above described modelling strategies; the OR could not be calculated for women.

There were 70 (25 pericardial and 45 TVT) occupationally exposed mesothelioma cases. In population-based studies, analyses by occupation are limited by the low prevalence of most specific jobs. As briefly reported in our paper, for purely descriptive purposes, the industrial activity of exposure (cases may have multiple exposures), were construction (22 exposures, 7 and 15 for pericardial and TVT mesotheliomas, respectively), steel mills and other metal working industries (4 and 11), textile industries (2 and 3), and agriculture (2 and 5); other sectors had lower exposure frequencies. The absence of industries like asbestos-cement production, shipbuilding and railway carriages production/repair should not be surprising and had already been observed (7). In the Italian multicenter cohort study of asbestos workers (8), given the person-years of observation accrued by workers employed in these industries and gender- and site-specific crude incidence rates, approximately 0.1 case of pericardial and 0.2 of TVT mesothelioma would have been expected from 1970 to 2010. Even increasing ten-fold such figures to account for higher occupational risks among these workers would not change much.

Asbestos exposure in agriculture has been repeatedly discussed in ReNaM reports (9: pages 70, 73, 128, 164 and 205). Exposure opportunities included the presence of asbestos in wine production, reuse of hessian bags previously containing asbestos, or construction and maintenance of rural buildings. Similarly, mesothelioma cases and agricultural workers exposed to asbestos have been noted in France (10).

In conclusion, the additional analyses we performed according to Mezei et al’s suggestions confirm the association between asbestos exposure and pericardial and TVT mesothelioma, supporting the causal role of asbestos for all mesotheliomas. ReNaM’s continuing surveillance system with national coverage is a precious platform for launching analytical studies on pleural and extra pleural mesothelioma.
